# Genes associated with fitness and disease severity in the pan-genome of mastitis-associated *Escherichia coli*

**DOI:** 10.3389/fmicb.2024.1452007

**Published:** 2024-08-29

**Authors:** Michael A. Olson, Caz Cullimore, Weston D. Hutchison, Aleksander Grimsrud, Diego Nobrega, Jeroen De Buck, Herman W. Barkema, Eric Wilson, Brett E. Pickett, David L. Erickson

**Affiliations:** ^1^Department of Microbiology and Molecular Biology, Brigham Young University, Provo, UT, United States; ^2^Faculty of Veterinary Medicine, University of Calgary, Calgary, AB, Canada

**Keywords:** mastitis, *Escherichia coli*, GWAS, chitinase, ExPEC

## Abstract

**Introduction:**

Bovine mastitis caused by *Escherichia coli* compromises animal health and inflicts substantial product losses in dairy farming. It may manifest as subclinical through severe acute disease and can be transient or persistent in nature. Little is known about bacterial factors that impact clinical outcomes or allow some strains to outcompete others in the mammary gland (MG) environment. Mastitis-associated *E. coli* (MAEC) may have distinctive characteristics which may contribute to the varied nature of the disease. Given their high levels of intraspecies genetic variability, virulence factors of commonly used MAEC model strains may not be relevant to all members of this group.

**Methods:**

In this study, we sequenced the genomes of 96 MAEC strains isolated from cattle with clinical mastitis (CM). We utilized clinical severity data to perform genome-wide association studies to identify accessory genes associated with strains isolated from mild or severe CM, or with high or low competitive fitness during *in vivo* competition assays. Genes associated with mastitis pathogens or commensal strains isolated from bovine sources were also identified.

**Results:**

A type-2 secretion system (T2SS) and a chitinase (ChiA) exported by this system were strongly associated with pathogenic isolates compared with commensal strains. Deletion of *chiA* from MAEC isolates decreased their adherence to cultured bovine mammary epithelial cells.

**Discussion:**

The increased fitness associated with strains possessing this gene may be due to better attachment in the MG. Overall, these results provide a much richer understanding of MAEC and suggest bacterial processes that may underlie the clinical diversity associated with mastitis and their adaptation to this unique environment.

## Introduction

Bovine mastitis often results from bacterial infection. Mastitis-associated *E. coli* (MAEC), abundant in the dairy environment, are the most important cause of this disease. These bacteria cycle between the bovine digestive tract and soils and beddings of stalls, from which they may gain access to the mammary gland (MG) via the teat canal. Once established within the MG, MAEC can induce a range of clinical presentations. Subclinical mastitis is usually defined as increased somatic cell counts in milk and transient inflammation caused by cytokine release. MAEC infections are typically cleared rapidly without complication or need for antibiotic intervention (Roberson et al., 2004; Fuenzalida and Ruegg, 2019). Conversely, severe clinical mastitis (CM) can damage the MG through sustained inflammation and high bacterial loads. These cows often suffer permanent udder damage, and the bacteria occasionally disseminate beyond the MG leading to sepsis (Wenz et al., 2001; Suojala et al., 2013). Some mastitis cases are characterized by mild acute disease followed by extended periods of chronic or recurrent infections. Occasionally MAEC strains gain access through the teat canal to the MG during the non-lactating period. Symptoms of CM develop shortly after the next lactation begins, which also tends toward chronic infections (Bradley and Green, 2000; Lippolis et al., 2014).

Features that distinguish MAEC from other *E. coli* strains have been difficult to identify and remain incompletely understood. Although they may come from any of the diverse *E. coli* lineages, MAEC strains often fall within *E. coli* phylogroups A and B1, which are also the most common phylogroups of commensal strains. Nevertheless, commensal strains are unable to cause acute clinical or chronic mastitis (Blum et al., 2015), suggesting that there are fundamental differences between commensals and MAEC strains that have yet to be discovered. MAEC frequently belong to sequence types (MLST) 10, 58, 95, and 1125 (Kempf et al., 2016; Leimbach et al., 2017; Blum and Leitner, 2013; Nuesch-Inderbinen et al., 2019; Freitag et al., 2017; Keane, 2016) but this also does not distinguish them from other strains. Genes predicted to be associated with MAEC have been examined in numerous PCR-based surveys as well as more extensive genomic studies (Blum et al., 2015; Nuesch-Inderbinen et al., 2019; Jamali et al., 2018; Ismail and Abutarbush, 2020; Lippolis et al., 2018).

Phenotypes distinctive of MAEC strains include relatively robust resistance to the complement system and greater motility than other *E. coli* (Lippolis et al., 2018; Guerra et al., 2020; Lippolis et al., 2016). The ferric dicitrate transport system encoded by the *fecABCDE* genes is also highly expressed and much more consistently found in MAEC genomes compared with other *E. coli* (Jung et al., 2021; Lin et al., 1999). Previously, we conducted a functional genetic screen using transposon insertion sequencing to identify the fitness factors of a single MAEC strain (Olson et al., 2018). This work demonstrated that the *fec* genes are needed to colonize lactating mouse MGs and implicated the high-affinity zinc transport system and several genes involved in other metabolic pathways in fitness in MGs. However, different genes may be required for fitness in other MAEC strain backgrounds. A more thorough understanding of MAEC genomics may help identify strains capable of causing mastitis from the varied strains found in agricultural settings, as well as those more likely to cause severe CM.

Mastitis severity depends on several host factors that contribute to disease outcome. For instance, the stage of lactation when the MG becomes infected has a strong influence on whether the infecting bacteria are efficiently cleared. At the time of parturition and earlier stages of lactation, cows are more prone to severe CM, which has been attributed to immunological dysfunction of neutrophils and lymphocytes during this time (Burvenich et al., 2007; Sordillo, 2005; Vangroenweghe et al., 2005; Hill, 1981; Cai et al., 1994; Burvenich et al., 1994).

In addition to host factors, bacterial factors may also influence CM severity (Guerra et al., 2020; Lehtolainen et al., 2003; Wenz et al., 2006). Some MAEC strains carry virulence factors often found in extraintestinal pathogenic *E. coli* (ExPEC) such as toxins, siderophores, capsules and adhesins (Olson et al., 2021). For the most part, the influence of these virulence factors during intramammary infections has not been determined. Thus far, the only trait with a demonstrated association with CM severity is swarming motility, which is higher in MAEC strains isolated from severe CM cases than those strains isolated from mild and moderate CM (Guerra et al., 2020). Gene expression comparisons of MAEC isolates from transient infections and persistent CM also demonstrated that flagella gene expression and motility are generally higher in the persistent CM strains (Lippolis et al., 2018). Persistent strains are also more resistant to serum complement and express the *fec* operon genes at higher levels than transient strains.

Previous genomic analyses have focused on identifying genes that are unique to MAEC isolates compared to non-pathogenic strains inhabiting the same niches or commensal strains belonging to the same phylogroups. These analyses have uncovered putative marker genes that could distinguish MAEC from other strains, including those that may function in niche-specific metabolic pathways, gene regulation, and virulence (Jung et al., 2021; Goldstone et al., 2016). In this study, we sequenced 96 MAEC genomes and implemented a comparative genomics approach to uncover genes more likely to be present in strains isolated from cattle with mild or severe CM. We then extended this analysis to identify genes associated with either mastitis isolates or commensal *E. coli* strains from cattle. We employed mouse infection and milk growth assays to separate MAEC strains with higher or lower fitness to identify genes that are associated with these phenotypes.

## Materials and methods

### Bacterial strains and growth conditions

*E. coli* strains M22 through M117 (Supplementary Dataset 1) were isolated from individual quarter milk samples from cattle with clinical mastitis as part of the Canadian National Cohort of Dairy Farms as previously described (Reyher et al., 2011). In brief, 89 herds across Canada (Alberta, Ontario, Quebec, and the Maritime provinces Prince Edward Island, New Brunswick, and Nova Scotia) were selected to be representative of their respective province in terms of housing type, bulk tank somatic cell count, cattle breed, and milking schedule, and were followed from February 2007 to December 2008. At the time of collection, farmers evaluated the clinical signs presented in each affected animal and assigned a clinical score, as follows: mastitis score 1 (mild = abnormal milk only), mastitis score 2 (moderate = abnormal milk and local inflammation signs), or mastitis score 3 (severe = abnormal milk, local inflammation and systemic clinical signs) (Sears and McCarthy, 2003). From this collection (Dufour et al., 2019), strains isolated from cows in the middle to late stages of their lactation cycle were selected in order to focus on bacterial differences and minimize the effect that early lactation has on mastitis severity (Hyvonen et al., 2010). Selection was also designed to include isolates from different herds and provinces. Six strains isolated from CM cases in the United States were also included in this study (Supplementary Dataset 1). Strains M3, M6, M9, M11, and M12 were previously isolated from quarter milk samples of CM cases (Olson et al., 2018). Clinical severity data were not available for these isolates. Strain G1 was supplied by Jennifer Wilson (Jersey Girls, Jerome ID) and was isolated from a cow with severe, gangrenous mastitis that necessitated culling of the animal.

The *E. coli* strains obtained from the mastitis pathogen culture collection (Dufour et al., 2019) were verified phenotypically by colony morphology on MacConkey agar (Difco, United States) plates and confirmed by whole-genome sequencing. Bacteria were routinely grown in Luria-Bertani (LB) medium at 37°C. For milk cultures, whole, unpasteurized cow’s milk was obtained from a local supplier and used immediately or stored at −80°C until use. To determine growth yields of individual MAEC isolates, bacteria from overnight LB cultures were added to 100 μL milk to a concentration of 10^3^ CFU/mL in a 96-well format. Plates were incubated without shaking at 37°C. A sample was immediately removed (*T* = 0), serially diluted and plated on MacConkey agar to determine the starting concentration. Bacterial concentrations were also measured at 4 h and 8 h post-inoculation. The change in CFU/ml at 4 h and 8 h relative to *T* = 0 was calculated for three biological replicates for each strain.

### Genome sequencing, assembly, and annotation

Total DNA was isolated from MAEC strains using a ZR Fungal/Bacterial DNA MiniPrep kit (Zymoresearch). DNA sequencing libraries were prepared using the Illumina Nextera DNA Library Prep kit as previously described (Baym et al., 2015). DNA libraries were sequenced by Genewiz, Inc. (South 330Plainfield, NJ), and Illumina paired-end reads of 150 bp were generated on a MiSeq with version 2 chemistry. Quality control, contig assembly, *in silico* determination of phylogroup, multi-locus sequence typing, and GrapeTree analysis were performed within EnteroBase (Zhou et al., 2020; Zhou et al., 2018) and genomes were annotated with Prokka (version 1.14.6) (Seemann, 2014). GrapeTree analysis was performed using the Achtman 7 Gene MLST scheme (*adk*, *fumC*, *gyrB*, *icd*, *mdh*, *purA*, *recA*) and the MSTree V2 algorithm. Accession numbers for genome assemblies are found in Supplementary Dataset 1 and are publicly available at Enterobase.^1^

### Core and accessory genome determination, alignment, phylogenetic trees, and pan-genome analysis

PIRATE (version 1.0.4 with default parameters) was used to perform a pan-genome analysis on all the GFF annotation files for all MAEC genomes and the results were then converted to the ROARY gene presence/absence format. The SNPs of all genes that comprised the core genome were concatenated in the same linear order prior to reconstructing a maximum-likelihood phylogenetic tree using IQ-Tree, and the subsequent tree was visualized using Interactive Tree of Life (Letunic and Bork, 2019; Minh et al., 2013; Nguyen et al., 2015). For the IQ-Tree phylogenetic reconstruction, ModelFinder (Kalyaanamoorthy et al., 2017) was employed to select the generalized time reversible model (GTR + R10), and an ultrafast bootstrap approximation (UFboot) with 1,000 bootstrap replicates was used. The PIRATE output was then input into the SCOARY (version 1.6.16 with default parameters) tool (Brynildsrud et al., 2016), which predicts clusters of orthologous genes across the core and accessory genome. Specifically, SCOARY identified members of the accessory genome that had a statistically significant association between gene presence and the tested phenotype. For the severity analysis, we selected 90 isolates belonging to mastitis scores 1 or 3 (44 mild, 46 severe). SCOARY was also used to identify genes in the pan-genome associated with competitive fitness in milk and in mouse MGs (using barcoded strains, see below). For this analysis, the top and bottom 30% of strains for each condition were separated based on their competition index (CI) values (regardless of whether they came from mild or severe CM cases). To identify genes associated with pathogenic or commensal strains, 220 genomes for bovine commensal strains were downloaded from NCBI using “bos taurus commensal” with the *E. coli* species tag. Similarly, 188 MAEC genomes were downloaded from NCBI using “bovine mastitis” with the *E. coli* species tag or from (Alawneh et al., 2020) using the python downloading programs at: https://github.com/SomeoneNamedCaz/E.-Coli-genome-analysis. SCOARY input files consisted of a gene absence/presence Rtab file generated by PIRATE and a custom trait file which assigned a discrete phenotype for each strain.

### Hierarchical clustering analysis

The MD Anderson Cancer Center Next-Generation Clustered Heat Map (NG-CHM) builder was used for hierarchical clustering^2^ based on the ExPEC virulence gene carriage. The Euclidian distance and single-linkage methods were used to generate the clusters.

### Detection of plasmids, antimicrobial resistance (AMR), and virulence genes

The PlasmidFinder 2.1 database at the Center for Genomic Epidemiology^3^ was used to detect and type plasmids found in the MAEC strains in this study (Supplementary Dataset 1). Assembled reads for each strain were searched using the most recent Enterobacteriaceae plasmid database using 90% minimum identity and 60% minimum length coverage cutoffs. Incompatibility (Inc) groups that were found in each strain were recorded. Each Inc. group was counted individually even when multiple Inc. groups were detected in a single strain. ABRicate software^4^ was used to find genes implicated in AMR from the Resfinder database (Zankari et al., 2012). Virulence genes were identified using the VirulenceFinder 2.0 program^5^ using 90% minimum identify and 60% minimum length cutoffs.

### Development of barcoded plasmids and barcode sequencing

The low copy pACYC184 plasmid was modified by designing PCR primers incorporating a partial Illumina adapter flanking 12 random nucleotides and eliminating the tetracycline resistance gene, yielding a 2,102 bp product (Supplementary Figure 1). The left primer incorporated the random sequences and the first Illumina Truseq adapter, and both primers included SalI overhang sequences. The PCR product was digested with SalI, ligated and transformed into chemically competent *E. coli* DH5α. Ninety-six unique plasmids were isolated, and their barcode sequence determined by Sanger sequencing. These plasmids were then transformed into individual MAEC isolates. Four strains were unable to be transformed because of pre-existing chloramphenicol resistance, leaving 92 strains that were successfully transformed. An inoculum was prepared by growing each strain individually in LB to an A_600_ = 1.0, mixing 20 μL of each strain, together, and diluting the mixture to a final concentration of 5 × 10^6^ CFU/ml in PBS. This inoculum was frozen for use in competition tests and was sequenced as the input library.

The input library was grown in duplicate in LB broth and in whole unpasteurized cow milk (pooled from healthy cows from a local commercial supplier) for 8 h. The input library was also injected into lactating mouse MGs (see below). After growth in LB, milk and MG infections, plasmids were isolated from the bacterial population in each sample. The barcodes were amplified using primers that also added the remainder of the Illumina adapter as well as sample-specific identifying sequence. Illumina paired-end reads of 150 bp were generated on MiSeq version 2 sequencer by Genewiz, Inc. (South Plainfield, NJ) and CD-Genomics, Inc. (Shirly, NY). A custom grep function was used to identify and count barcodes for each strain from the sequence reads. Fitness scores were calculated as the number of reads for each barcode in an output sample as a proportion of the total reads (all barcodes) in that sample, divided by the ratio of that same barcode to the total reads in the inoculum (input) library.

### Mouse infections

Lactating CD-1 IGS mice between 9 and 12 weeks of age and 10 to 11 days postpartum were infected as previously described (Olson et al., 2018). Protocol 16–0302 was reviewed and approved by the Institutional Animal Care and Use Committee of Brigham Young University. Briefly, a 50 μL volume of bacteria containing 500 CFU of each strain for total of ~50,000 CFU was suspended in phosphate-buffered saline (PBS). The inoculum was injected directly through the teat canal into the ductal network of the 4th left and 4th right MGs of five individual mice using a 33-gage needle with a beveled end. Pups were removed for 1–2 h after injections and then reunited with the mother and allowed to nurse normally. Mice were euthanized 24 h post infection and each individual gland was separately homogenized in 1 mL PBS. The tissue homogenate was added to LB broth containing chloramphenicol (10 μg/mL) to recover the bacteria and isolate plasmid DNA. Sequencing of each MG sample (as described above) was successful for nine of the 10 individual glands. The barcode frequencies in each gland (*n* = 9) were determined and used to determine competitive fitness of each strain.

### Deletion and complementation of *chiA*

An allelic exchange plasmid was created using the pAX1 plasmid (Wiles et al., 2018). Upstream and downstream regions (500 bp) of *chiA* in strain M45 were amplified, stitched together using overlap extension, and inserted into the SalI and AvrII sites of the pAX1 plasmid. The resulting plasmid was transformed via electroporation into the donor *E. coli* strain MFDλpir. Integration of the suicide plasmid to create merodiploid recipients and excision to produce unmarked deletions of *chiA* in each of the strains was performed as described (Wiles et al., 2018). Complementation of *chiA* gene was done by amplifying *chiA* from strain M45 including 300 bp upstream to include the putative promoter and ligating the resulting product into pJET1.2. The resulting plasmid pWH01 was verified by sequencing and transformed via electroporation into each deletion mutant.

### MAC-T cell culture and media

Bovine mammary alveolar epithelial cells (MAC-T cells) were generously provided by Dr. Janos Zempleni (University of Nebraska-Lincoln). They were grown in T-75 flasks with 40% (v/v) Dulbecco’s Modified Eagle Medium (DMEM), 40% (v/v) Ham’s F12 Medium, and 10% (v/v) bovine serum (FBS). FBS was heat-inactivated prior to use in media by incubating in a 56°C water bath for 30 min with periodical mixing. This was supplemented with 5 μg/mL bovine insulin, 1 μg/mL hydrocortisone, 23 mM HEPES buffer, 2.2 g/L sodium bicarbonate, and 40 mM L-glutamine. Penicillin, streptomycin (100 U/mL and 100 μg/mL, respectively) and amphotericin B (2.5 μg/mL) were added into the media for routine growth. MAC-T cells were grown at 37°C and 5% CO_2_ (v/v).

### Adhesion assays

Adhesion was measured as previously described (Almeida et al., 1996) with modifications. MAC-T cells were seeded into a 12-well plate and grown to ≥95% confluency. The density of epithelial cells was determined by trypan blue exclusion and counting using Cell Counter model R1 automated cell counter (Olympus). Approximately 6×10^5^ cells were present in each well. The number of cells and viability did not vary throughout the assays as determined by trypan blue exclusion. On the day of each assay, spent media was removed from each well and the cells were washed 3 times with 1 mL of sterile PBS to remove residual antibiotics from media. Media without antibiotics was added into each well and cells were allowed to incubate for ≥2 h prior to inoculation with bacteria.

Overnight cultures of bacteria were diluted in sterile PBS to an OD_600_ of 0.5. Bacteria were added to a multiplicity of infection (MOI) of 10:1. The 12-well plates were then centrifuged at 300 x g for 5 min at room temperature to synchronize contact of bacteria with the MAC-T cells. Plates were then incubated at 37°C and 5% CO_2_ (v/v) for 1 h. Following incubation, media was aspirated and wells washed 3x with 1 mL of sterile PBS to dislodge non-and weakly adhered bacteria. Triton X-100 (500 μL, 0.1%) in PBS was then added into each well and incubated at room temperature for 5 min to lyse cells. The resulting suspension of bacteria was then homogenized, serially diluted and plated on LB agar overnight and CFUs were counted.

### Statistical analyses

Statistical analyses were carried out using Prism9 (GraphPad) or SCOARY. A *p* < 0.05 was considered statistically significant. For genome-wide association (GWAS) studies, Fisher’s exact test was used to determine significance for each gene. Associations of the Inc. group with a strain type (mild or severe CM) were assessed using Fisher’s exact test. Distribution of AMR genes between mild and severe CM isolates was compared using the Mann–Whitney *T*-test. Correlations between fitness scores were determined by Spearman rank correlation analysis or Mann–Whitney *T*-test. Differences in adherence to MAC-T cells by wild-type, *chiA* mutant, or complemented strains were analyzed using a one-way ANOVA.

## Results

### Bacterial growth in milk is not associated with clinical mastitis severity

The ability to efficiently utilize the nutritional components present in milk and thus grow rapidly may be a determining factor for some strains ability to colonize MGs successfully. To determine if there is a link between *in vitro* growth in milk and CM severity, we compared the growth of a subset of mild and severe MAEC strains in whole unpasteurized cow’s milk (Supplementary Figure 2). Replication was measured after 4 or 8 h of growth. MAEC strains varied widely in their growth yields, with some strains replicating 100-fold within 4 h and up to 100,000-fold by 8 h, whereas others did not increase. However, replication was not different between strains isolated from mild and severe CM at either time point.

### Genome analysis of mild and severe clinical mastitis isolates

Complete genome sequences for 90 MAEC strains (46 severe and 44 mild CM isolates) were assembled and annotated (Supplementary Dataset 1). From these annotated sequences, we identified a total of 15,395 unique genes representing the pan-genome. Of these, 3,177 (20.6%) were considered core genes (>98% prevalence), 263 (1.7%) were considered soft core genes (95–98% prevalence), 1,523 were considered shell genes (15–95% prevalence), and 10,432 (67.8%) were considered cloud genes (<15% prevalence) (Supplementary Table 1).

**Figure 1:**
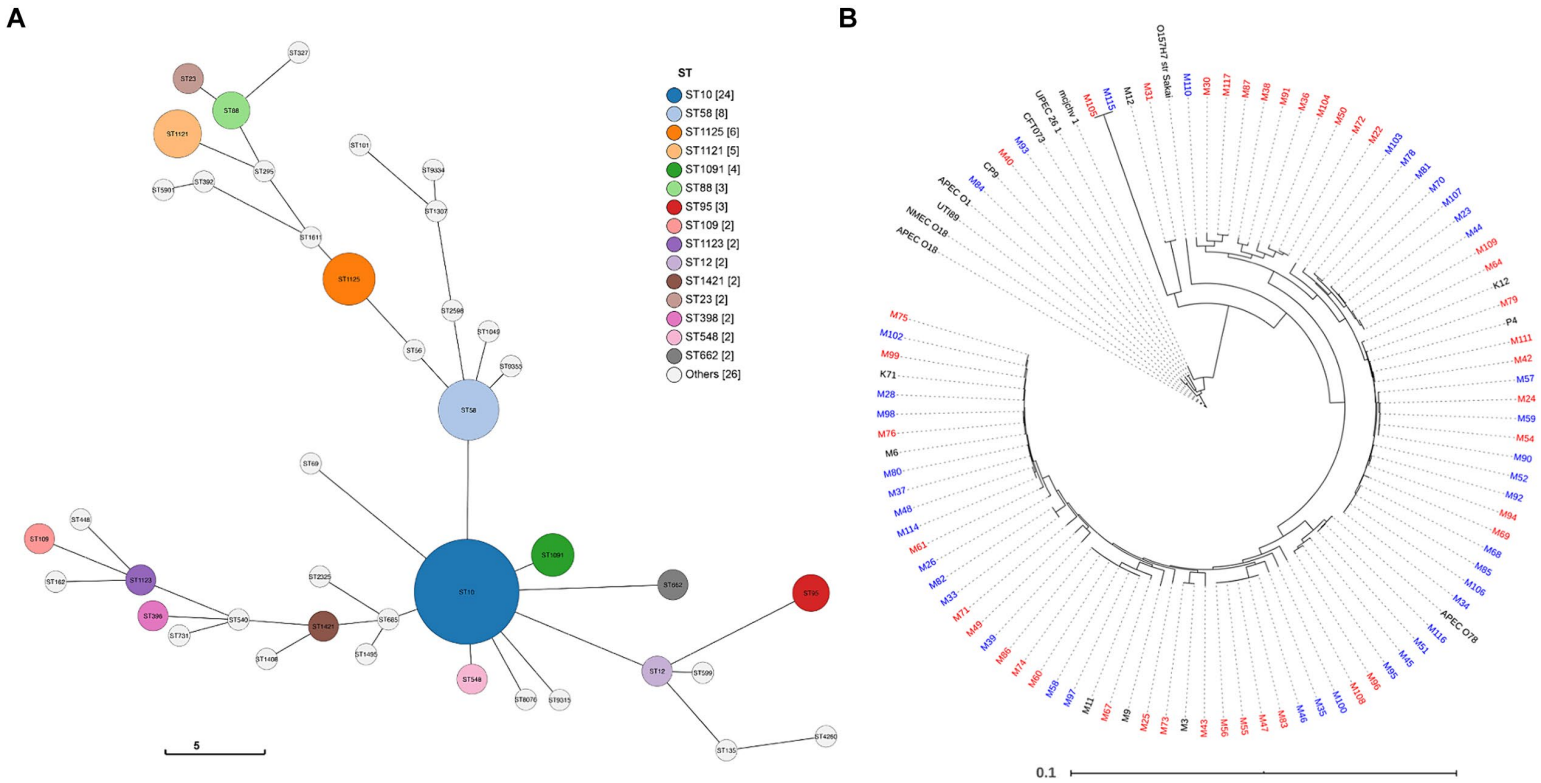
Genome comparison of MAEC strains. **(A)** Sequence type (ST) prediction using the GrapeTree function with the MSTree V2 algorithm to visualize strain relatedness (Zhou et al., 2020; Zhou et al., 2018). Overall, 30 unique STs were detected. The legend displays STs represented more than once in the population (node diameter scaled to frequency of detection). **(B)** Core genome relatedness among strains isolated from severe mastitis or mild mastitis. Strains isolated from mild CM cases are denoted in blue and severe CM in red. Strains labeled black were included as references or did not have clinical severity data and were not part of the analysis.

*In silico* phylogroup analysis demonstrated that most strains belong to phylogroups A and B1. Multi-locus sequence typing based on seven house-keeping genes was also used to more precisely assess the phylogenetic backgrounds of the MAEC strains (Supplementary Dataset 1). A total of 30 different STs were identified, with many STs being represented by only one strain each. In our study, ST10, ST58, ST1121 and ST1125 were the most abundant, representing 45% of the total strains (Figure 1A). MLST groupings did not correlate with mild or severe CM.

To explore whether CM severity can be predominantly attributed to differences in the core genome, a phylogenetic tree was constructed based on the core genome sequence alignments. In this analysis, several ExPEC strain, one enterohemorrhagic strain, and additional MAEC strains without clinical severity data were included as references (Figure 1B). The phylogenetic tree reveals the high diversity of MAEC strains. Compared to the relatively tight clustering of other ExPEC, the MAEC strains belong to a broad range of backgrounds, including some closely related to other ExPEC strains. Of 10 strains that clustered closely with other ExPEC strains, nine were isolated from severe CM and one from mild CM. However, there was no consistent clustering into clades based on CM severity. There was also no association between the core genome and the geographical region where the strains were isolated (Supplementary Figure 3).

**Figure 2:**
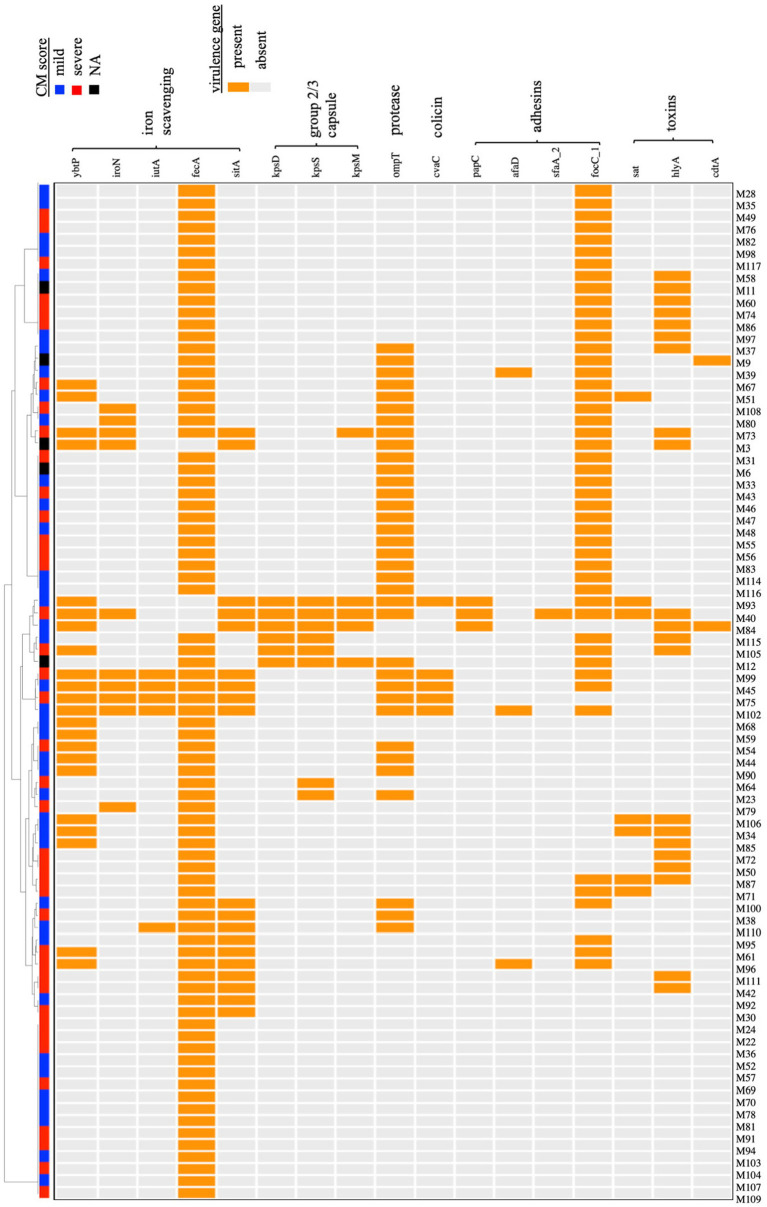
Hierarchical clustering of MAEC strains based on the carriage of virulence genes associated with the ExPEC phenotype. A dendogram was built based on the presence or absence of each gene. Both mild and severe CM isolates were present in each clade, and no relationship between CM severity and particular combinations of virulence genes was detected. NA-CM severity score not available.

ExPEC are difficult to define based on any single or group of virulence genes. However, they often carry genes related to iron acquisition (yersiniabactin *ybtP*, aerobactin *iutA*, salmochelin *iroN* siderophores, ferric citrate *fecA*, and *sit* ferrous iron transport), group 2 or 3 capsules (*kps*), adhesive and invasive factors [P fimbriae; *papC,* S fimbriae; *sfaA*, *focC*, afimbiral adhesins (*afaD*), and toxins (*sat*, *hlyA*, *cdtA*)] (Johnson and Stell, 2000; Johnson et al., 2003; Royer et al., 2023; Welch, 2016; Sarowska et al., 2019). These genes were detected in several of the MAEC genomes (Figure 2). Mean number of virulence genes carried by each strain was 3.32 ± 2.15 or 3.17 ± 2.23 for mild and severe isolates, respectively, demonstrating that simple abundance of ExPEC virulence genes is not predictive of CM severity. For example, strain M93 (mild isolate) carries 10 of these ExPEC virulence genes. As CM severity could be associated with specific combinations of virulence genes, hierarchical clustering was then performed based on the presence or absence of each gene (Figure 2). This analysis demonstrated no apparent clustering of strains based on CM severity and ExPEC virulence gene content.

**Figure 3:**
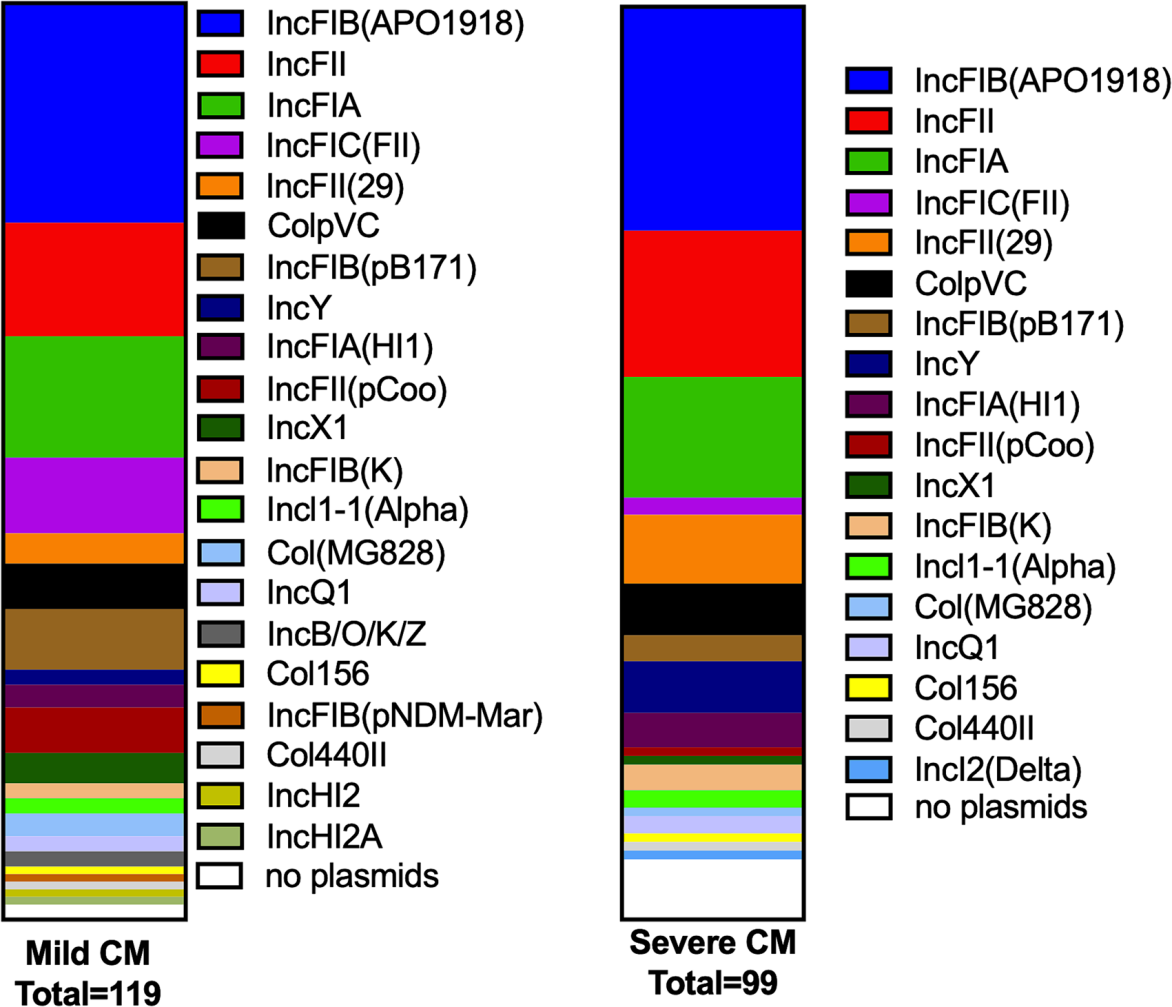
Plasmid replicons (Inc types) found in mild and severe CM strains. The number of plasmid replicon genes detected in mild and severe strains is presented as a proportion of the total for each group. More unique replication genes were detected in the mild CM strains compared with severe CM strains, whereas more of the severe CM strains contained no detectable Inc. types than the mild CM strains.

Plasmids contribute to bacterial diversity such as that demonstrated by our study population, in part because they can contribute to homologous or non-homologous recombination. Plasmids also frequently carry specific fitness or AMR genes, which could influence CM severity. Incompatibility (Inc) typing was performed for each of the MAEC strains, and 22 different Inc. types were detected, suggesting a wide diversity of plasmid content (Figure 3 and Supplementary Dataset 1). Multiple Inc. types were frequently detected in the same strain, suggesting the possibility of hybrid plasmids. When compared to the severe CM strains, more Inc. types were detected among the mild CM strains and were more frequent overall, whereas 15.2% of severe CM isolates and 4.5% of mild CM isolates did not carry any plasmids (Figure 3). IncF1B (APO1918) was the most abundant Inc. type and was detected in the majority of mild and severe CM strains (65.9 and 56.5% respectively).

Carriage of AMR genes among mild and severe CM strains was also assessed *in silico* by searching a repository of AMR genes. This analysis demonstrated that 12 strains carried one or more genes conferring resistance to aminoglycosides, beta-lactams, anti-folates, macrolides, phenicols, and tetracyclines (Supplementary Table 2). Aminoglycoside and anti-folate resistance genes were the most abundant. Notably, two severe CM isolates (M79 and M96) had nine AMR genes each. However, the distribution of AMR genes was not significantly different between mild and severe CM isolates (*p* = 0.6029).

### Genes associated with clinical mastitis severity

Next, we analyzed each gene in the pan-genome and scored it according to the association with mild or severe CM phenotypes. Each gene with an apparent association with the phenotype was reanalyzed, incorporating information about the phylogenetic structure to implicate genes associated with CM severity. Using a *p*-value cutoff of 0.05, we detected 25 genes that were associated with severe CM and 79 genes associated with mild CM (Supplementary Dataset 1 and Table 1). Among those associated with severe CM were two genes likely involved in O-antigen or capsule biosynthesis (*wbpI* and *capD*) and several genes involved in producing Yad fimbriae. These genes were detected in eight strains isolated from severe CM cases and were not present in any strains from mild CM cases. Genes encoding fimbrial protein subunits YadM, YadL, YadK and YadN as well as the fimbrial usher HtrE and chaperone YadV were all positively associated with severe CM.

**Table 1:** Top genes* associated with MAEC isolated from mild or severe clinical mastitis (CM).

		Proportion of strains		
Gene name	Annotation	Severe CM	Mild CM	*P*-value
*Mild*				
*xerC_5*	Tyrosine recombinase XerC	0.429	0.756	2.96E-03
*xapA*	Purine nucleoside phosphorylase 2	0.667	0.902	1.05E-02
*xapB*	Xanthosine permease	0.667	0.902	1.05E-02
*pgaD*	Biofilm PGA synthesis protein PgaD	0.810	0.976	1.18E-02
*xerC_1*	Tyrosine recombinase XerC	0.048	0.244	1.31E-02
*hcaR_2*	Hca operon transcriptional activator HcaR	0.690	0.902	2.01E-02
*traA*	Pilin	0.476	0.732	2.20E-02
*Severe*				
*wbpI*	UDP-2,3-diacetamido-2,3-dideoxy-D-glucuronate 2-epimerase	0.190	0.000	5.36E-03
*capD*	UDP-glucose 4-epimerase	0.190	0.000	5.36E-03
*yadV*	Putative fimbrial chaperone YadV	0.190	0.000	5.36E-03
*yadM*	Putative fimbrial-like protein YadM	0.190	0.000	5.36E-03
*yadK*	Putative fimbrial-like protein YadK	0.190	0.000	5.36E-03
*htrE*	Outer membrane usher protein HtrE	0.190	0.000	5.36E-03
*cfaE*	CFA/I fimbrial subunit E	0.214	0.024	1.45E-02
*ygcB*	CRISPR-associated endonuclease/helicase Cas3	0.286	0.073	2.01E-02
*casB*	CRISPR system Cascade subunit CasB	0.286	0.073	2.01E-02
*hpcD*	5-carboxymethyl-2-hydroxymuconate Delta-isomerase	0.500	0.244	2.20E-02
*hscC_2*	Chaperone protein HscC	0.143	0.000	2.57E-02
*fliD*	Flagellar hook-associated protein 2	0.571	0.317	2.59E-02
*ygbF*	CRISPR-associated endoribonuclease Cas2	0.310	0.098	2.74E-02
*casE*	CRISPR system Cascade subunit CasE	0.310	0.098	2.74E-02
*casD*	CRISPR system Cascade subunit CasD	0.310	0.098	2.74E-02
*ygbT*	CRISPR-associated endonuclease Cas1	0.310	0.098	2.74E-02
*casC*	CRISPR system Cascade subunit CasC	0.310	0.098	2.74E-02
*yadL*	Putative fimbrial-like protein YadL	0.190	0.024	2.91E-02
*yadN*	Putative fimbrial-like protein YadN	0.190	0.024	2.91E-02

^*^Excluding hypothetical proteins.

Most genes associated with mild CM were hypothetical genes. However, genes for producing the biofilm PGA exopolysaccharide and several tyrosine recombinases were associated with mild CM. The complete *pgaABCD* operon was detected in nearly all (39 of 40) strains with a mild CM score whereas just *pgaD* was missing in eight strains from severe CM. Three tyrosine recombinase alleles (Blum and Leitner, 2013; Goldstone et al., 2016; Dufour et al., 2019) were associated with mild CM strains, while distinct alleles (Fuenzalida and Ruegg, 2019; Lippolis et al., 2016; Cai et al., 1994) were associated with severe CM strains (Table 1).

### Genes associated with mastitis vs. commensal strains

We conducted a complementary analysis of a larger publicly available set of *E. coli* genomes to identify genes that may distinguish commensal from mastitis-causing strains. These included strains isolated from the gastrointestinal tracts of cattle, which were designated as commensals. Likewise, publicly available genome sequences for all strains with an identifiable mastitis designation as well as strains from this study or published by Alawneh et al. (2020) were designated as pathogens. As expected, genes within the ferric dicitrate receptor (*fec*) operon were among the most strongly associated with the mastitis isolates. The *gadB* gene encoding the glutamate decarboxylase B enzyme was more often absent from the mastitis isolates (detected in 30.3% genomes) whereas the commensal strains typically contained *gadB* (84.5%). Genes encoding the accessory type II secretion system (*gsp*) and the linked *chiA* gene were also strongly associated with disease-causing strains (Table 2).

**Table 2:** Top genes* associated with mastitis or commensal bovine *E. coli* isolates.

		Proportion of strains		
Gene name	Annotation	Commensal	Mastitis	*P*-value
*Commensal*				
*gadB*	Glutamate decarboxylase B subunit	0.846	0.303	7.84E-35
*tufB*	Elongation factor Tu	0.865	0.420	3.11E-25
*cytR*	Cytidine repressor	0.765	0.415	6.53E-15
*Mastitis*				
*fecB*	Ferric dicitrate ABC transporter - periplasmic binding protein	0.360	0.878	1.07E-31
*fecD*	Ferric dicitrate ABC transporter - membrane subunit	0.354	0.872	1.33E-31
*fecE*	Ferric dicitrate ABC transporter - ATP binding subunit	0.354	0.872	1.33E-31
*fecI*	RNA polymerase sigma 19 factor	0.379	0.888	2.48E-31
*fecR*	Regulator for fec operon periplasmic	0.379	0.888	2.48E-31
*fecA*	Ferric citrate outer membrane porin FecA	0.379	0.888	2.48E-31
*fecC*	Ferric dicitrate ABC transporter - membrane subunit	0.360	0.872	4.61E-31
*yjhV*	KpLE2 phage-like element predicted protein	0.344	0.835	8.14E-28
*mqsA*	Antitoxin of the MqsRA toxin-antitoxin system and DNA-binding transcriptional repressor	0.273	0.644	5.53E-16
*yafN*	Antitoxin of the YafO-YafN toxin-antitoxin system	0.280	0.649	6.72E-16
*yafO*	Ribosome-dependent mRNA interferase toxin	0.273	0.638	1.23E-15
*mqsR*	mRNA interferase toxin of the MqsR-YgiT toxin-antitoxin system	0.273	0.638	1.23E-15
*ygiS*	Putative transporter subunit	0.328	0.691	3.16E-15
*ybfP*	Putative pectinase	0.251	0.590	
*gspO*	Type 4 prepilin-like proteins leader peptide-processing enzyme	0.289	0.633	6.79E-14
*gspK*	Putative type II secretion system protein K	0.292	0.633	8.86E-14
*gspA*	Putative general secretion pathway protein	0.292	0.633	8.86E-14
*gspF*	Putative type II secretion system protein F	0.292	0.633	8.86E-14
*gspH*	Putative type II secretion system protein H	0.292	0.633	8.86E-14
*chiA*	Putative bifunctional chitinase/lysozyme	0.292	0.633	8.86E-14
*gspM*	Putative type II secretion system protein M	0.292	0.633	8.86E-14
*gspJ*	Putative type II secretion system protein J	0.292	0.633	8.86E-14

*Excluding hypothetical proteins.

### Genes associated with fitness in milk and mammary glands

Next, we tested whether strains isolated from severe or mild CM have different fitness levels within the MG environment. We competed 92 MAEC strains against each other and used GWAS to identify genes associated with fitness. In these assays, pools of barcoded MAEC strains were grown in LB media, whole unpasteurized milk, and mouse MGs. Seventy-three bacterial strains were successfully recovered (sequencing failed for 19 barcodes and these strains were excluded from the analysis). From these 73 remaining strains, the abundance of each barcode was used to calculate the competition index (CI) as a measurement of fitness for each strain (Supplementary Dataset 1).

**Figure 4:**
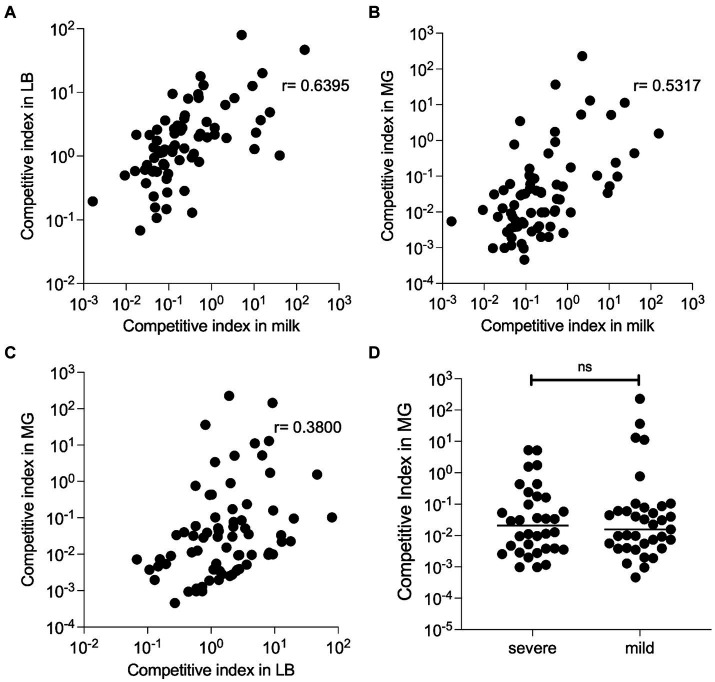
Competitive fitness of barcoded MAEC strains. All strains were inoculated together and grown in LB or unpasteurized cow’s milk (in duplicate) for 8 h, or in nine lactating MGs for 24 h. The bacteria were recovered, and their barcode plasmids were sequenced to determine their competitive indexes. **(A)** CI during *in vitro* growth in LB is strongly correlated with CI during growth in milk (*p* < 0.0001 by Spearman rank correlation test). Each dot represents a single MAEC strain plotted at the mean competition index in both environments. **(B)** A slightly weaker positive correlation was detected between CI in milk and MGs (*p* < 0.0001) and in **(C)** LB and MGs (*p* = 0.0008) by Spearman rank correlation test. **(D)** CI in mouse MG infections for MAEC strains isolated from mild or severe CM cases. No significant difference was detected in these groups Student’s *t*-test with Mann–Whitney correction (*p* = 0.904).

The CI scores that were calculated for each strain in duplicate LB and milk samples were highly consistent with each other (R^2^ = 0.99 and 0.94 respectively). The replicate samples for the nine MG infections were much more variable than in LB or milk. However, several strains consistently outcompeted others in mouse MGs (Supplementary Figure 4). For instance, strain M65 exhibited a mean CI of 225. The mean CI of each strain during growth in LB and milk, (Figure 4A), milk and MGs (Figure 4B), and LB and MGs (Figure 4C) were positively correlated. Correlation between CI milk and MGs was slightly stronger (*r* = 0.53) than between LB and MGs (*r* = 0.38). No association between the diagnosed CM severity (mild vs. severe) and fitness in mouse MGs was evident (Figure 4D).

Genes in the pan-genome that are associated with competitive fitness in milk and in mouse MGs were then identified. For this analysis, the top and bottom 30% of strains for each condition were separated based on their CI scores. Only five genes were positively associated with higher growth in milk, including a predicted inner membrane protein *yjeO,* three genes with unknown function, and an IS3 family transposase, *insK*. In mouse MGs, 38 genes were positively associated with increased competitive fitness (Supplementary Dataset 1). These included the bacterioferritin (*bfr*) and bacterioferritin-associated ferredoxin (*bfd*) genes that are involved in intracellular iron storage and mobilization. Notably, 14 of these genes are involved in the accessory type 2 secretion system found in two operons (*gspC-O* and *gspAB*) as well as the chitinase encoded by *chiA* (Table 3). The type 2 secretion system and *chiA* were also more frequently associated with mastitis-causing strains than with commensals (Table 2).

**Table 3:** Top genes* associated with MAEC fitness in mouse MG infections.

		Proportion of strains		
		Most fit	Least fit	*P-*value
*rpiB*	Ribose-5-phosphate isomerase B	0.812	0.313	3.85E-03
*bfd*	Bacterioferritin-associated ferredoxin	0.812	0.375	2.90E-02
*bfr*	Bacterioferritin	0.812	0.375	2.90E-02
*gspB*	Putative general secretion pathway protein B	0.812	0.375	2.90E-02
*gspC*	Putative type II secretion system protein C	0.812	0.375	2.90E-02
*gspA*	Putative general secretion pathway protein A	0.812	0.375	2.90E-02
*gspF*	Putative type II secretion system protein F	0.812	0.375	2.90E-02
*gspG*	Putative type II secretion system protein G	0.812	0.375	2.90E-02
*gspD*	Putative secretin GspD	0.812	0.375	2.90E-02
*gspE*	Putative type II secretion system protein E	0.812	0.375	2.90E-02
*gspJ*	Putative type II secretion system protein J	0.812	0.375	2.90E-02
*gspH*	Putative type II secretion system protein H	0.812	0.375	2.90E-02
*gspI*	Putative type II secretion system protein I	0.812	0.375	2.90E-02
*gspO*	Type 4 prepilin-like proteins leader peptide-processing enzyme	0.812	0.375	2.90E-02
*gspL*	Putative type II secretion system protein L	0.812	0.375	2.90E-02
*gspM*	Putative type II secretion system protein M	0.812	0.375	2.90E-02
*gspK*	Putative type II secretion system protein K	0.812	0.375	2.90E-02
*chiA*	putative bifunctional chitinase/lysozyme	0.812	0.375	2.90E-02
*alsB*	D-allose-binding periplasmic protein	0.812	0.375	2.90E-02

*Excluding hypothetical proteins.

These associations suggested that ChiA might promote fitness of pathogenic strains. As ChiA proteins are known to enhance adherence of other bacteria to host cells or tissues, the role of ChiA in adhesion to cultured mammary alveolar epithelial (MAC-T) cells was investigated. We chose to investigate three strains that were in our original study population that possess *chiA* (M45, M93, M111) as well as one additional strain that was not part of the original analysis (G1). M45 was among the top 30% most fit strains in MGs. M45 and M93 were isolated from mild cases of CM while M111 was isolated from a severe case. Strain G1 was isolated from a case of severe, gangrenous mastitis.

**Figure 5:**
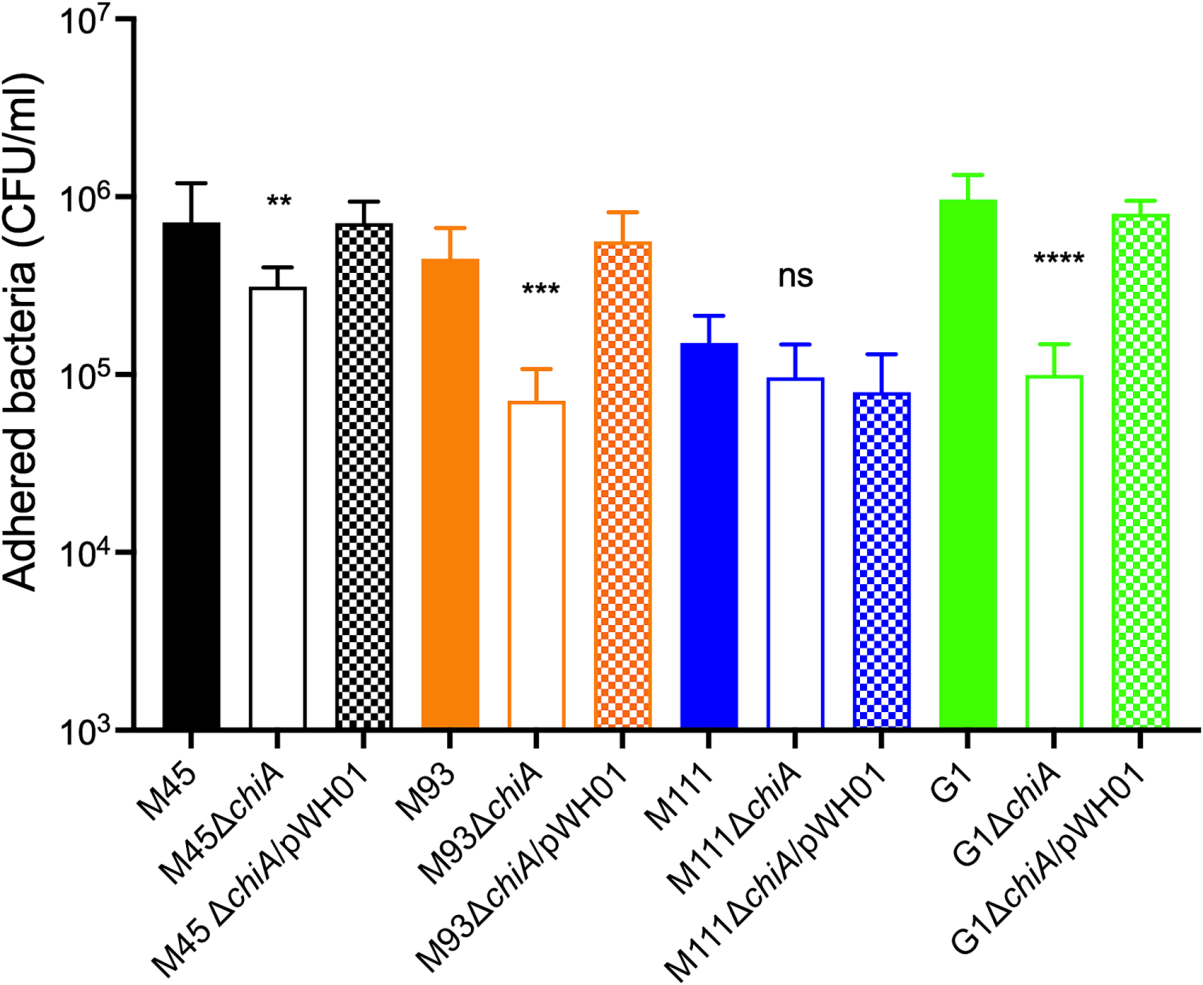
Role of ChiA in adherence of MAEC to mammary epithelial cells. Wild-type MAEC strains, their isogenic Δ*chiA* mutants, or the complemented mutant strains were tested for their ability to adhere to MAC-T cells at an MOI = 10. One-way ANOVA with Tukey’s correction was used to determine significant differences between the wild-type and each Δ*chiA* mutant (^**^p = 0.0082, ^***^p = 0.0003, ^****^p < 0.0001). These results are a representative experiment that was performed twice with six replicates per strain.

Wild type, *chiA* deletion mutants, or their complemented strains were added to MAC-T cells (MOI = 10) for 1 h and then the proportion of adherent cells measured. M45Δ*chiA* demonstrated an approximately 2-fold reduction compared to the wild-type parent (3.1 × 10^5^ vs. 7.2 × 10^5^ CFUs, respectively) in attachment (Figure 5). Attachment was restored upon reintroduction of *chiA* by plasmid complementation. A greater reduction was observed in M93 (>6-fold) between the wild type and mutant (Figure 5), which was also able to be complemented. The slight reduction in attachment of M111Δ*chiA* compared to the wild-type strain was not statistically significant (*p* = 0.06) and complementation did not change adherence of this strain (Figure 5). Deletion of *chiA* in G1 caused the largest decrease in attachment of all the MAEC strains we investigated. G1Δ*chiA* presented almost a 10-fold decrease when compared to the wild type as shown in Figure 5. Wild-type levels of adherence were restored upon plasmid complementation in G1.

## Discussion

The features of pathogenic *E. coli* that differentiate them from non-pathogens remain incompletely understood, as is the relationship between bacterial fitness and the clinical disease that occurs during infection. Several recent studies have employed genome-wide association tools paired with clinical or experimental data to identify accessory genes associated with bacterial virulence, niche adaptation, AMR, and environmental persistence (Bazinet, 2017; Maury et al., 2019; Gouliouris et al., 2018; Zong et al., 2018; He et al., 2018; Fritsch et al., 2019; Gori et al., 2020). In this study we sequenced 96 MAEC genomes, with the goal of identifying bacterial genes associated with differences in manifestation of bovine CM. We also identified genes associated with commensal strains in comparison with clinical bovine mastitis isolates. We developed a barcoding system where multiple strains can be tracked as they compete in different conditions. This allowed us to quantify differences in fitness in specific controlled conditions and use these differences to identify genes associated with high and low-fitness strains. This approach enabled the identification of novel genes that may influence bacterial growth in multiple host environments. Our study differs from most bacterial GWAS in that it combines clinical and mouse infection assay data to identify a gene that was validated by functional studies.

Our data confirm the diversity of MAEC strains (Figure 1) and reveals the large pan-genome associated with these bacteria. In previous genomic studies of MAEC strains, most were classified as ST10, ST23, ST58, ST88, or ST1125 in phylogroups A or B1, which is consistent with our findings (Leimbach et al., 2017; Blum and Leitner, 2013; Nuesch-Inderbinen et al., 2019). Within the accessory genome, we identified genes encoding adhesive and extracellular matrix structures associated with strains isolated from mastitis cases diagnosed as either mild or severe (Table 1). This includes the Yad fimbriae, which were positively associated with mastitis severity, and conversely, the PGA biofilm exopolysaccharide was negatively associated with mastitis severity. Yad fimbriae are also enriched among ExPEC strains of avian and human origin and are thought to promote adherence (Ren et al., 2016; Wurpel et al., 2013; Dziva et al., 2013; Verma et al., 2016; Larsonneur et al., 2016; Elpers and Hensel, 2020). Sustained inflammation in the MG is a main trigger for severe CM and bacterial factors that promote adherence would likely increase immune detection and signaling, leading to phagocytic cell recruitment. Alternatively, PGA may lessen CM severity by promoting biofilm formation, shielding inflammatory molecules on the surface of the bacteria and/or reducing invasion into epithelial cells.

MAEC are typically excluded from discussion of ExPEC strains more broadly. ExPEC exact an outsized disease burden in both humans and animals and are difficult to distinguish reliably from other *E. coli*. We did find several instances of MAEC strains possessing genes with demonstrated roles in ExPEC virulence (Figure 2). Although these virulence factors were not associated with CM severity generally, it does not rule out the possibility that they contribute to the virulence of individual strains in MGs, as we have previously demonstrated for the capsule and zinc uptake gene clusters of strain M12 (Olson et al., 2018; Olson et al., 2021). Furthermore, additional factors continue to be recognized that contribute to ExPEC infections, and these may also be selected by the dairy environment and enriched in MAEC strains. The ferric dicitrate iron acquisition system is one such example (Frick-Cheng et al., 2022). While their role in intramammary infection has been established, they were recently demonstrated to enhance ExPEC urovirulence, suggesting that the *fec* system may be a factor in zoonotic spread of *E. coli* since it promotes fitness in multiple hosts and tissue types (Frick-Cheng et al., 2022). The presence of these classic and newly appreciated ExPEC virulence genes in MAEC strains suggests that they occasionally infect humans and cause bloodstream or urinary tract infections.

We identified many different Inc. plasmid groups in our MAEC strains and found that mild CM strains had significantly more Inc. groups than severe CM strains (Figure 3). However, we did not investigate whether any virulence or AMR genes were carried on these plasmids as is frequently the case. The majority of the plasmids we detected belong to the IncF family which are usually conjugative (Douarre et al., 2020), illustrating their potential to spread resistance in these populations. AMR is a significant health and environmental concern. However, only 12 strains carried one or more AMR genes. Two strains carried genes that confer resistance to six different classes of antimicrobials. Carriage of AMR genes did not appear to be associated with disease severity.

Unsurprisingly, our results indicate that those strains that are highly competitive *in vitro* tend to outcompete other strains *in vivo*, due to more rapid growth, direct antibacterial antagonism, or both (Figure 4). Interestingly, the correlation between competitive fitness in milk with MG infections than was slightly stronger than the correlation between fitness in LB and MG, suggesting that the ability to utilize nutrients or resist antimicrobial substances found in milk contributes to growth in lactating MGs. However, the lack of correlation we observed between bacterial fitness and CM severity (Figure 4D) illustrates that successful pathogens may replicate to high numbers without triggering deleterious responses in the host. Similarly, we observed no relationship between *in vitro* growth rates of individual strains in milk with CM severity (Supplementary Figure 2), which has also been reported by other researchers (Kornalijnslijper et al., 2004).

The *chiA* gene encoding a putative chitinase/chitin-binding protein was enriched in MAEC with higher fitness during mouse MG infections. This gene, along with the type II secretion system linked with it, was also associated with pathogenicity in the larger cohort of bovine strains. However, they were not associated with CM severity. In the non-pathogenic K12 strain, transcription of this type II secretion system is normally repressed by the Hns protein, and the full *gsp* locus is needed for proper *chiA* secretion (Francetic et al., 2000). ChiA has been implicated in the virulence of some adherent/invasive *E. coli* strains that cause colitis. This is not due to their chitinolytic activity, but rather because of chitin-binding domains that are found in the N-terminus. These domains mediate binding to chitinase-3-like-1 (CHI3L1), which is expressed on the surface of intestinal epithelial cells, leading to subsequent invasion of the bacteria (Low et al., 2013; Mizoguchi, 2006). Interestingly, expression of host chitin-like proteins is also induced by some bacterial pathogens (Lee et al., 2011). CHI3L1 regulates innate immune defenses against *Streptococcus pneumoniae* and *Pseudomonas aeruginosa* lung infections through inhibition of caspase-1-dependent macrophage pyroptosis (Dela Cruz et al., 2012; Marion et al., 2016). Conversely, CHI3L1 expressed by intestinal epithelial cells during inflammatory bowel disease helps facilitate enteric bacterial infection.

Recently, CHI3L1 was also found in the milk secretions of quarters with bovine coliform mastitis (Breyne et al., 2018). CHI3L1 gene expression is also increased in mouse MGs following *E. coli* infection. In knockout mice lacking CHI3L1, bacterial growth is not affected, but the influx of neutrophils into the lumen of the infected gland is reduced (Breyne et al., 2018). It also promotes increased proliferation of mammary epithelial cells and reduces apoptosis (Anand et al., 2016). In the absence of CHI3L1, migration, maturation, and activation of macrophages is significantly impaired (Hughes et al., 2012; He et al., 2013). It seems likely that some MAEC strains may bind to CHI3L1 via ChiA, which could promote bacterial attachment and invasion and suppression of its inflammatory functions. In this way, bacterial fitness may be increased while limiting disease severity.

Alternatively, it is possible that ChiA contributes to infection through glycosidase activity, independently of its binding to host proteins. *Salmonella* chitinases modify glycans present in the extracellular matrix, uncovering mannose residues present on the epithelial surface and making them available for attachment through type I fimbriae (Chandra et al., 2022; Devlin et al., 2022). They also increase survival inside phagocytes by dampening the expression of host antimicrobial responses in dendritic cells and macrophages (Chandra et al., 2022). ChiA may play similar roles in MAEC colonization of MGs.

Our study has several limitations. First, the barcoding plasmid that we used to track mixed populations of bacteria may not behave identically in each strain’s unique genetic background. Although the plasmid has only one coding sequence for chloramphenicol resistance and is unlikely to affect gene expression broadly, the presence of the plasmid may interfere with stability of other native plasmids, which we have not examined. This may influence the fitness of these bacteria in unexpected ways. Secondly, competitive fitness of MAEC strains may be most relevant in natural environments in the presence of many other bacterial species other than *E. coli*. The ability to outcompete other *E. coli* strains may be less critical for MAEC than their ability to defend themselves against other diverse bacteria in the cattle GI tract, in soil, or ascending the MG teat canal. Future work should test what genes contribute to fitness in these environments and whether they can explain why some MAEC strains are more common, particularly those STs that we and others have identified. Finally, the results of our study may have been influenced by random factors such as when CM was diagnosed and scored. For example, a case that was detected early may have been diagnosed as mild whereas if diagnosed a few hours later may have been scored as moderate or severe. Diagnosis of CM is also inherently subjective, and the criteria may have been interpreted differently by individual farmers.

The wide range of severity and clinical presentation of MG infections by MAEC is impressive, and more studies directly characterizing the putative virulence factors of these strains are required. In this study, we have identified new accessory genes that could play a role in host specificity of these bacteria and influence disease outcomes. The role of ChiA in colonizing MGs as well as its functions in other ExPEC strains deserves further study, including its potential role in immune suppression and interaction with host structures. The ability of some MAEC strains to cross host species barriers and colonize different tissues underscores the importance of better understanding the diversity among this group of bacteria.

## Data Availability

The datasets presented in this study can be found in online repositories. The names of the repository/repositories and accession number(s) can be found in the article/Supplementary material.

## Footnotes

^1^ https://enterobase.warwick.ac.uk

^2^ https://build.ngchm.net/NGCHM-web-builder/

^3^ http://genepi.food.dtu.dk/resfinder

^4^ https://github.com/tseemann/abricate

^5^ https://cge.food.dtu.dk/services/VirulenceFinder
